# Evaluation of the Effect of Elite Jojoba Lines on the Chemical Properties of their Seed Oil

**DOI:** 10.3390/molecules27123904

**Published:** 2022-06-17

**Authors:** Nahla A. Awad, Mohamed Eliraq, Emad H. El-Bassel, Ahmed S. M. Ismail, Yasser S. G. Abd El-Aziz, Mohamed S. Gawish, Reda M. Y. Zewail, Rokayya Sami, Ebtihal Khojah, Uguru Hilary, Maalem H. Al-Moalem, Khaled Sayed-Ahmed

**Affiliations:** 1 Agriculture Research Center, Horticulture Research Institute, Giza 12619, Egypt; nohawad@yahoo.com (N.A.A.); shakour13@yahoo.com (M.E.); emadelbassel@yahoo.com (E.H.E.-B.); asm8019@yahoo.com (A.S.M.I.); y.ezat@yahoo.com (Y.S.G.A.E.-A.); 2 Pomology Department, Faculty of Agriculture, Damietta University, Damietta 34512, Egypt; 3 Botany Department, Faculty of Agriculture, Benha University, Benha 13736, Egypt; reda.zewail@fagr.bu.edu.eg; 4 Department of Food Science and Nutrition, College of Sciences, Taif University, P.O. Box 11099, Taif 21944, Saudi Arabia; eykhojah@tu.edu.sa; 5 Department of Agricultural Engineering, Delta State University of Science and Technology, Ozoro 334111, Nigeria; erobo2011@gmail.com; 6 Department of Home Ecomomics, College of Education–Delam, Prince Sattam Bin Abdulaziz University, P.O. Box 173, Alkharj 11942, Saudi Arabia; m.almoalem@psau.edu.sa; 7 Department of Agricultural Chemistry, Faculty of Agriculture, Damietta University, Damietta 34512, Egypt; drkhaled_1@du.edu.eg

**Keywords:** jojoba oil, chemical composition, peroxide value, iodine number, fatty acids

## Abstract

Jojoba oil (JO) extracted from seeds has outstanding properties, including anti-inflammatory, antioxidant, and antibacterial activities, and can be stored forlong periodsof time. The unique properties of jojoba oil depend on its chemical composition; therefore, the effect of the jojoba genotype on the chemical properties and active components of the seed oil was evaluated in this study. Oil samples were collected from 15 elite Egyptian jojoba lines. The chemical composition, such as moisture, crude fiber, crude oil, ash, and crude protein of elite lines’ seeds was determined to investigate the variation among them based on the jojoba genotype. In addition, the iodine value was obtained to measure the degree of jojoba oil unsaturation, whereas the peroxide number was determined as an indicator of the damage level in jojoba oil. Fatty acid composition was studied to compare elite jojoba lines. Fatty acid profiles varied significantly depending on the jojoba genotype. Gadoleic acid exhibited the highest percentage value (67.85–75.50%) in the extracted jojoba oil, followed by erucic acid (12.60–14.81%) and oleic acid (7.86–10.99%). The iodine value, peroxide number, and fatty acid composition of the tested elite jojoba lines were compared withthose reported by the International Jojoba Export Council (IJEC). The results showed that the chemical properties of jojoba oils varied significantly, depending on the jojoba genotype.

## 1. Introduction

Jojoba (*Simmondsia chinensis* (Link) C.K. Schneid) belongs tothe family *Simmondsiaceae* and is native to southwestern United States and northern Mexico. It is an evergreen shrub that has attracted considerable interest owing to its unique properties [1]. Jojoba seeds are rich in crude oil (up to 50%) and are considered an economical source ofwax esters, usually known as jojoba oil (JO) [2]. Jojoba shrubs are highly resistant to drought, enabling them to produce crops without the need for large amounts of water comparedtotraditional crops. Jojoba production can be significantly improved by systematic germplasm collection, variety trials, clonal evaluation, and cultivar selection [3]. JO is considered an ideal recompensefor sperm whale oil, which enhances its use in various industrial applications such as the pharmaceutical industry [4]. JO has great benefits for human skin; therefore, it can be used in several cosmetic products such as moisturizers, face washes, hair shine enhancers, foot softeners, and makeup removers, owing to its dermatological characteristics [5]. In addition, JO can be used as a carrier for sensitive oxidizing substances, such as vitamin A and essential oils. Jojoba liquid wax is helpful for stabilizing penicillin products [6,7]. JO usually exhibits a high iodine value in the range-82–86 g, indicating the high content of unsaturated fatty acids found in JO. Therefore, JO can improve blood cholesterol levels, stabilize heart rhythms, and ease inflammation, in addition to several other medical roles [8].High levels of unsaturated fatty acids found in JO can increase antioxidant mechanisms and minimize mitochondrial depolarization [9]. The oxidative stability of JO indicates that it is a stable oil and suitable for storage conditions. JO has a lower peroxide value than other natural oils because of its high oxidative stability and resistance to rancidity as well as its relatively high content of natural antioxidants such as α, γ, and δ tocopherols [10,11]. The fatty acid composition of JO included both saturated and unsaturated fatty acids. Unsaturated fatty acids, such as eicosanoic, docosenoic, oleic, linoleic, and linolenic acids, represent the major fatty acids (up to approximately 97%), and saturated fatty acids, including palmitic, pentadecanoic, myristic, and lauric acids, occupy a lower percentage than unsaturated fatty acids [12,13]. Based on the above, JO has attracted the interest of researchers conducting several qualitative and quantitative analyses to study its chemical properties [14].

The chemical properties and composition of JO vary depending on the environmental conditions, including climate, specific environment (ecotype), soil type, andgenetic factors [15]. Some studies have reported that the chemical properties of JO are significantly affected by genetic variability in jojoba. Therefore, the evaluation of genetic variability may be key to improving jojoba yield and properties in the near future [16]. Several studies have beenreported on cultivated Egyptian jojoba shrubs [17,18,19,20,21], however, few have investigated the chemical properties of JO and its chemical composition.

Hence, the main aim of this study was to evaluate the effect of genetic variability on the chemical composition of Egyptian jojoba seeds and the potential variations in the chemical characteristics of JO of elite female jojoba lines selected by Nahla et al. [22,23]. In this respect, the fatty acidcomposition, iodine number, and peroxide value of JO were determined to investigate the significant differences amongelite jojoba lines. Additionally, the chemical constituents of the jojoba seeds were analyzed.

## 2. Results and Discussion

### 2.1. Chemical Composition of the JO

The chemical composition of the seeds of elite jojoba lines was determined to study the effect of jojoba genotype on the chemical constituents of the seeds. Notably, the crude oil, moisture, ash, crude protein, and crude fiber contents varied depending on the jojoba genotype properties, as listed in Table 1. The results obtained from the chemical composition analysis revealed that S_3_, S_6_, and S_14_ showed higher oil percentage values of 48.11, 48.05, and 48.15, respectively, compared to the other tested lines. In contrast, S_2_, S_4_, and S_9_ had lower oil contents of approximately 42%. Gad et al. reported similar results and found that oil weight represented approximately 52% of the total weight of jojoba seeds. JO, the only unsaturated liquid wax extracted from plant sources in large quantities, has a chemical composition similar to sperm whale oil [24]. This similarity enhances the applicability of JO as an inexpensive replacement for sperm whale oil. The total protein content of jojoba seeds was determined by a colorimetric method using a Biuret solution. S_7_ had the highest protein content (12.55%), whereas S_1_ seeds had the lowest protein content (11.93%) compared to the other lines. Cappillino et al. and Mohamed [25,26] determined the total protein content of jojoba seeds and found similar protein contents of 15.2 and 14.72%, respectively. Additionally, the moisture percentage in the seeds of the different elite jojoba lines was determined to study the potential variation among these lines based on their chemical constituents. In this respect, there was a notable change in the moisture content rangingfrom 3.4 to 5.2%. The crude fiber content of jojoba seeds differed significantly as the change in jojoba lines was in the range of 9.94–17.74%.

### 2.2. Peroxide and Iodine Values

The peroxide value was determined based on the iodine contentformed by the reaction of oil peroxides with iodide ions. The iodine number was obtained to determine the amount of unsaturated fatty acids in the JO [27]. The iodine and peroxide values of JO also varied depending on the jojoba strain, as listed in Table 2. The obtained results revealed that S_13_ showed the highest iodine value of 94.75, whereas S_9_ exhibited the lowest value (82.28), indicating that the total amount of unsaturated fatty acids differed significantly with the variation in jojoba lines. JO has a high iodine number, revealing that it is helpful in fighting infections and has anti-inflammatory properties. Unsaturated fatty acids are considered beneficial because they can improve blood cholesterol levels, stabilize heart rhythms, and ease inflammation. In addition, they play several other medical roles [8]. Oils extracted from S_13_, S_14_, and S_15_ seeds are preferable for pharmaceutical applications because of their high iodine values of 94.75, 94.02, and 91.78, respectively [24].

The practical utility of the peroxide value is to evaluate oxidative rancidity and is considered an important indicator of the oil degradation level. The peroxide value was expressed as milliequivalents of peroxide per kg of oil (mEq/kg). The peroxide values ranged from 0.25 to 1.19 mEq/kg, as listed in Table 2. In addition, oils from different elite jojoba lines showed low peroxide values, indicating high stability, minimum oxidation levels, and applicability for long storage periods. The oxidative stability of JO and its high resistance to rancidity may be due to the presence of natural antioxidants, such as α, γ, and δ tocopherols in the oil [10]. The S_13_ and S_14_ oils were more stable than other elite lines. They exhibited lower peroxide values of 0.25 and 0.27 mEq/kg, respectively. In general, all tested elite lines showed peroxide values less than the standard, as shown in Table 2. Bergfeld et al. reported similar results and found that the wax of Jojoba exhibited a low peroxide value (0–0.5 mEq/kg) when stored at room temperature (20 °C) in the dark for several months [11]. The iodine and peroxide values listed in Table 2 show significant differences because of the variation in genotypic properties and geographical occurrence, as shown in Table 2.

### 2.3. Fatty Acid Profile

Pure waxes, including wax esters, alcohols, a few free fatty acids, and hydrocarbons, represent approximately 98% of the total chemical composition of JO, as well as vitamins, sterols, and a few triglyceride esters. Based on this, JO is widely known as liquid wax rather than as fat or oil [29].

The data presented in Table 3 show the common fatty acid contents in JO. It was observed obviously that the oils of all the elite jojoba lines were rich in unsaturated fatty acids. The results revealed that the fatty acid contents determined in JO differed significantly among elite lines.

Gadoleic acid (C20:1), also known as gondoic acid, is an unsaturated omega-9 fatty acid. It representsthe main fatty acid in the oil of jojoba seeds and exhibitsthe highest percentage value, ranging from 67.85% to 75.50%, compared to the other fatty acids, as shown in Table 3. It is similar to human sebum, which acquires high absorption in the human skin and can thereforemoisturize the skin without a greasy effect [30]. The high content of gadoleic acid in the oils of elite jojoba lines enhances their applicability in the pharmaceutical and cosmetic industries. In addition, the presence of gadoleic acid, an unsaturated fatty acid, at high concentrations confirmed the high iodine values determined for different elite jojoba lines.

Erucic acid was observed at high concentrations in the oils of the tested elite Jojoba lines and was in the range of 12.60–14.81% of the total fatty acids. It is also an unsaturated omega-9 fatty acid biosynthesized by oleic acid elongation using oleoyl-coenzyme A and malonyl-CoA [31]. Chronic exposure to erucic acid can cause cardiac lesions, myocardial lipidosis, and hepatic steatosis. Therefore, its concentration in edible oils is restricted to limited levels. However, a mixture of oleic acid and erucic acid is usually utilized as a dietary treatment for adrenoleukodystrophy, without cytotoxicity [32]. In this respect, the presence of oleic acid at high concentrations (7.86–10.99%) may decrease the cytotoxicity of erucic acid found in the oil of jojoba seeds.

S_3_, S_14_, and S_15_ had low percentages of erucic acid (12.6–12.84%) and high concentrations of oleic acid, ranging from 9.88 to 9.95%,which minimizes their cytotoxicity and augments their biological activity. In contrast, S_9_ and S_12_ oils had low amounts (7.86 and 8.41%) of oleic acid and the highest erucic acid percentage values of 14.81 and 14.73%, respectively, compared to the other elite jojoba lines.

The relative distribution of fatty acids in the oils of all elite lines containing palmitic acid (C16:0) ranged from 0.20% to 3.18%. Similar results were reported by Bilin et al., who found that the concentration of palmitic acid was in the range of 1–1.98% [19]. All jojoba lines were in the standard range, except S_4_, which exhibited the highest palmitic content (3.18%) compared to other elite jojoba lines. Palmitic acid has numerous benefits, such as improved skin health, anti-inflammatory effects, and enhanced metabolic health. However, at higher concentrations, it may have harmful effects on human health [33].

Palmitoleic acid was not detected in JO, except for S_13_, which exhibited only a low content (0.26%) of palmitoleic acid. Additionally, stearic acid was observed only in S_13_, S_14_, and S_15_ at concentrations of 0.16, 0.66, and 0.37, respectively. Vaccenic acid is a natural trans-fatty acid thatis considered the main trans-fatty acid present in human milk [34]. Vaccinic acid (C18:1) was found in low amounts in the oils of the jojoba seeds of the various elite lines, ranging from 0.72 to 1.62%. Linoleic acid (C18:2) was found in the oils of S_2_, S_3_, S_6_, S_7_, S_13_, S_14_, and S_15_ with low content (0.20–1.17%) varied based on the jojoba strain. In contrast, it was not detected in oils from the seeds of other elite lines. The linolenic acid (C18:3) content was in the range of 0.15–0.36%. It was not detected in all jojoba lines and was found at low concentrations only in the S_1_, S_2_, S_4_, S_13_, S_14_, and S_15_ oils, as listed in Table 3.

Based on the above, the fatty acid profiles of all tested jojoba lines varied depending on the jojoba strain genotype. Gadolic acid was the main fatty acid present in all jojoba seed oils, followed by erucic and oleic acids.

## 3. Materials and Methods

### 3.1. Plant Source

Jojoba seeds were harvested in August from different fifteen elite jojoba female lines selected, depending on morphological and seed characteristics as well as yield, pollen viability, and molecular identification using the Inter Simple Sequence Repeats (ISSR) marker, as described by Nahla et al. [23]. Consequently, this study aimed to investigate the effect of their varied genotypic properties on the chemical composition and chemical characteristics of their oils as a result of the aforementioned variations among these lines. The seeds were stored in the specific store at ambient temperature (22 ± 2 °C) with 30% humidity for one month untiloil extraction. Jojoba lines were collected from various regions (El-Kasasin and Cairo-Alexandria Desert road) in Egypt; these 15 lines were described in the previous searches with codes (C1, C2, C3, C4, C5, C7, C10, C16, C18, C19, C21, C22, C25, K7, K15) [23] and symbolled here with codes (S_1_ to S_15_) as listed in Table 4.

### 3.2. Methods

#### 3.2.1. Chemical Composition of Jojoba Seeds

##### Moisture Content

The moisture content of the seeds was analyzed by drying the seed samples of different elite jojoba lines at 70 °C until the weight was stable [35]. Three replicates were used for this analysis.

##### Oil Extraction

Jojoba seeds of elite lines were weighed and dried at 60 °C until a constant weight was achieved. The crude oil content of the seeds was determined using the Soxhlet method as described by Agarwal et al. [36]. JO was extracted using a Soxhlet apparatus with petroleum ether (bp 30–40 °C) as the solvent. The extracted oil was then allowed to evaporate at room temperature for 24–48 h. The defatted seeds were dried and weighed to obtain the crude oil content.

##### Protein Content

Total nitrogen in the jojoba seeds was estimated using the Micro-Kjeldahl method [37]. Defatted seeds were weighed and added to Kjeldahl flasks. They were then digested until the color of the solution changed to colorless and then allowed to cool. Boric acid (10 mL; 2%) and three drops of modified indicator, including a mixture of methyl red and methylene blue solutions, were added to a 50 mL conical flask. The samples were then attached to a Kjeldahl apparatus using a condenser tip immersed in boric acid. Sodium hydroxide (10 mL, 40%) was added to each sample and heated to boiling temperature. The ammonia obtained was then determined by titration with hydrochloric acid.

##### Crude Fiber Content

The crude fiber content was determined in the defatted jojoba seeds by treating the samples with 1.25% H_2_SO_4_. They were then subjected to additional treatment with NaOH (1.25%) solution. The crude fiber content in jojoba seeds of various elite lines was determined as described in the AOAC method [35].

##### Ash Content

The ash content of jojoba seeds of elite lines was determined according to the method described by AOAC [35].

#### 3.2.2. Evaluation of the Chemical Parameters of JO

##### Peroxide Value

The peroxide number was determined as an indicator of JO quality, which increased with a decrease in the peroxide number. Briefly, JO (5 g) was placed in a conical flask with a stopper. Subsequently, 30 mL of the solvent was added, followed by gentle shaking until the oil was completely dissolved. The air inside the conical flask was gently replaced with nitrogen to remove residual oxygen. Saturated potassium iodide (0.5 mL) was then added and the flask was immediately sealed with gentle shaking for approximately one minute. The conical flask was kept in the dark at room temperature (20–25 °C). Thirty milliliters of distilled water were added, and the container was sealed under stirring. The mixture was titrated with sodium thiosulfate (0.01 mol/L). The peroxide number was expressed as milliequivalents of peroxide per kilogram of oil (mEq/kg) [38].

##### Iodine Value

JO (0.1 g) was added to a 250 mL conical flask with a stopper. Subsequently, 20 mL of carbon tetrachloride was added to the flask and sealed. After sonication, 25 mL of Hanus solution was added and the mixture was shaken for 1 min. The mixture was allowed to stand in the dark at room temperature (20 °C) for 30 min. Then, 10 mL of potassium iodide (15%) and 100 mL of water were added, and the mixture was shaken for 30 s. The obtained mixture was titrated against sodium thiosulfate (0.1 mol/L) until the color changed from yellow to colorless to determine the iodine value [39].

##### Fatty Acids Composition

The fatty acids composition was determined using the GC–MS system (GC–MS QP-2010 Plus, Shimadzu, Kyoto, Japan) with an Rtx-5 MS column with dimensions of 30 m × 0.25 mm (0.25 μm film thickness). First, the oven temperature was set to 50 °C for 3 min and then gradually increased to 280 °C for 30 min. An aliquot of 1.0 μL of the tested oil sample was injected for fatty acid determination. Helium was used as the carrier gas at a flow rate of 1.2 mL per min. The injector and mass transfer line temperatures were adjusted to 270 and 280 °C, respectively. The split ratio was set at 10 in the analysis. In addition, the ionization mass of the spectroscopic analysis was performed at 70 eV. The mass spectrum was recorded in the range 40–600 m/z for 42 min. The data were obtained using GC–MS post-run software [36].

#### 3.2.3. Statistical Analysis

The obtained data were statistically analyzed using the Costat system version 6.311 (CoHort software, Monterey, CA, USA). First, all comparisons were subjected to variance analysis (ANOVA). Then, the standard deviation was calculated in addition to significant variations among all line means that were obtained from Duncan’s new range test at *p* = 0.05 (Duncan, 1955) [40,41,42]. Three replicates were used for each analysis.

## 4. Conclusions

In this study, fifteen elite Egyptian jojoba lines were selected to study the effects of genotype variety on the chemical properties of jojoba oil. There were significant variations in the chemical composition of jojoba seeds and the chemical properties of JO among elite jojoba lines as a result of the genotypic variation.

JO has a high iodine value of up to 94.75 and low peroxide value, indicating health benefits, high stability, minimum oxidation level, and applicability for long storage periods. The oils of all elite jojoba lines were rich in unsaturated fatty acids. However, fatty acid composition varies according to the genetic variety. Gadoleic acid was the main fatty acid, followed by erucic and oleic acids.

## Figures and Tables

**Table 1:** Chemical constituents of the seeds of elite jojoba lines.

Lines	Crude Oil %	Moisture %	Crude Protein %	Crude Fiber %	Ash %
S_1_	45.38 ± 0.42 d	4.20 ± 0.19 de	11.66 ± 0.39 h	11.08 ± 0.21 h	1.76 ± 0.10 ab
S_2_	42.13 ± 0.71 f	4.25 ± 0.21 de	12.53 ± 0.29 ef	17.55 ± 0.37 a	1.45 ± 0.08 d
S_3_	48.11 ± 0.69 a	3.93 ± 0.19 ef	13.47 ± 0.30 b	17.74 ± 0.32 a	1.82 ± 0.21 ab
S_4_	41.97 ± 0.64 f	4.07 ± 0.12 e	12.04 ± 0.35 g	11.25 ± 0.35 h	1.84 ± 0.16 ab
S_5_	47.34 ± 0.52 b	3.90 ± 0.20 ef	12.10 ± 0.25 g	9.94 ± 0.36 i	1.56 ± 0.11 cd
S_6_	48.05 ± 0.56 a	4.74 ± 0.12 b	13.12 ± 0.21 cd	16.15 ± 0.20 b	1.63 ± 0.06 bc
S_7_	43.21 ± 0.54 e	5.26 ± 0.15 a	12.55 ± 0.23 e	17.71 ± 0.22 a	1.78 ± 0.10 bc
S_8_	46.21 ± 0.46 c	4.02 ± 0.14 e	13.42 ± 0.28 bc	12.22 ± 0.27 g	1.68 ± 0.14 ab
S_9_	42.07 ± 0.57 f	3.71 ± 0.18 fg	12.10 ± 0.30 g	13.12 ± 0.28 f	1.83 ± 0.13 ab
S_10_	48.15 ± 0.60 a	3.70 ± 0.10 fg	12.14 ± 0.26 g	13.31 ± 0.35 ef	1.97 ± 0.11 a
S_11_	45.17 ± 0.54 d	3.43 ± 0.20 g	11.93 ± 0.33 gh	14.51 ± 0.22 c	1.75 ± 0.10 ab
S_12_	45.04 ± 0.63 d	4.65 ± 0.11 bc	13.89 ± 0.37 a	13.57 ± 0.20 e	1.86 ± 0.11 ab
S_13_	46.87 ± 0.48 b	3.62 ± 0.12 g	11.94 ± 0.33 gh	14.29 ± 0.27 d	1.72 ± 0.12 ab
S_14_	48.31 ± 0.64 a	4.34 ± 0.13 cd	12.98 ± 0.21 d	14.16 ± 0.34 d	1.91 ± 0.10 ab
S_15_	43.45 ± 0.38 e	4.60 ± 0.15 bc	12.23 ± 0.25 fg	10.20 ± 0.29 i	1.86 ± 0.09 ab

The data represented as the mean ± standard deviation (SD) of three replicates. a–i means with the same letter within the same column are not significantly different according to Duncan’s multiple range test (*p* = 0.05).

**Table 2:** Peroxide and iodine values of elite jojoba lines.

Lines	Iodine Value (Gram)	Peroxide Value mEq/Kg
S_1_	82.61 ± 0.59 fg	0.99 ± 0.09 ab
S_2_	84.38 ± 0.98 cd	0.50 ± 0.08 f
S_3_	84.22 ± 0.79 cde	0.71 ± 0.11 de
S_4_	83.66 d ± 0.32 ef	0.65 ± 0.07 ef
S_5_	82.68 ± 0.47 fg	0.92 ± 0.09 bc
S_6_	83.60 ± 0.30 fg	0.95 ± 0.13 bc
S_7_	84.94 ± 0.79 c	1.07 ± 0.07 ab
S_8_	83.20 ± 0.32 efg	0.73 ± 0.08 e
S_9_	82.28 ± 0.48 g	0.87 ± 0.10 cd
S_10_	84.00 ± 0.33 cde	1.04 ± 0.09 ab
S_11_	83.91 ± 0.79 cde	0.49 ± 0.08 f
S_12_	83.67 ± 0.38 def	1.16 ± 0.10 a
S_13_	94.75 ± 0.29 a	0.25 ± 0.07 g
S_14_	94.02 ± 1.02 a	0.27 ± 0.05 g
S_15_	91.78 ± 0.49 b	1.19 ± 0.07 a
IJEC Standard, 1998–AOCS [28]	82–87/100 (AOCS Cd 1–25)	2.0 (max) (AOCS Cd 8–53)

The data are presented as the mean ± standard deviation (SD) of three replicates. a–g means with the same letter within the same column are not significantly different according to Duncan’s multiple range test (*p* = 0.05).

**Table 3:** Fatty acids contents of jojoba oils of the elite lines.

Lines	Palmitic Acid C16:0	Plamitioleic Acid C16:1	Stearic Acid C18:0	Vaccinic Acid C18:1	Oleic Acid C18:1	Linoleic Acid C18:2	Linolenic Acid C18:3	Gadoleic Acid C20:1	Erucic Acid C22:1
S_1_	1.26 ± 0.04 de	0.00 ± 0.00 b	0.00 ± 0.00 d	1.14 ± 0.04 c	9.30 ± 0.11 d	0.00 ± 0.03 g	0.15 ± 0.01 e	75.50 ± 0.47 a	13.04 ± 0.22 h
S_2_	1.15 ± 0.05 ef	0.00 ± 0.00 b	0.00 ± 0.00 d	0.77 ± 0.07 gh	9.35 ± 0.04 d	0.69 ± 0.01 c	0.29 ± 0.02 c	74.10 ± 0.42 cd	13.59 ± 0.10 f
S_3_	1.22 ± 0.02 e	0.00 ± 0.00 b	0.00 ± 0.00 d	0.73 ± 0.04 h	9.95 ± 0.17 b	0.38 ± 0.00 d	0.00 ± 0.00 f	74.90 ± 0.41 ab	12.84 ± 0.10 hi
S_4_	3.18 ± 0.20 a	0.00 ± 0.00 b	0.00 ± 0.00 d	0.75 ± 0.06 gh	10.99 ± 0.15 a	0.00 ± 0.00 g	0.36 ± 0.02 a	67.85 ± 0.32 h	13.35 ± 0.15 fg
S_5_	0.98 ± 0.10 fg	0.00 ± 0.00 b	0.00 ± 0.00 d	0.72 ± 0.02 h	9.52 ± 0.14 cd	0.00 ± 0.03 g	0.00 ± 0.00 f	74.45 ± 0.38 bc	14.05 ± 0.18 de
S_6_	1.16 ± 0.11 ef	0.00 ± 0.00 b	0.00 ± 0.00 d	0.88 ± 0.05 f	9.66 ± 0.07 c	0.23 ± 0.01 ef	0.00 ± 0.00 f	72.93 ± 0.49 e	14.35 ± 0.09 cd
S_7_	1.16 ± 0.13 ef	0.00 ± 0.00 b	0.00 ± 0.00 d	0.80 ± 0.03 g	10.00 ± 0.14 b	0.24 ± 0.00 e	0.00 ± 0.00 f	72.53 ± 0.21 h	14.45 ± 0.23 bc
S_8_	0.97 ± 0.11 fg	0.00 ± 0.00 b	0.00 ± 0.00 d	0.86 ± 0.04 f	8.78 ± 0.14 e	0.00 ± 0.00 g	0.00 ± 0.00 f	73.93 ± 0.41 cd	14.27 ± 0.14 cde
S_9_	1.22 ± 0.11 e	0.00 ± 0.00 b	0.00 ± 0.00 d	1.04 ± 0.04 d	8.41 ± 0.09 f	0.00 ± 0.01 g	0.00 ± 0.00 f	73.76 ± 0.42 d	14.81 ± 0.11 a
S_10_	0.20 ± 0.04 h	0.00 ± 0.00 b	0.00 ± 0.00 d	1.62 ± 0.02 a	9.91 ± 0.16 b	0.20 ± 0.00 f	0.00 ± 0.00 f	72.95 ± 0.19 e	13.61 ± 0.26 f
S_11_	0.89 ± 0.05 g	0.00 ± 0.00 b	0.00 ± 0.00 d	0.86 ± 0.01 f	8.09 ± 0.07 g	0.00 ± 0.00 g	0.00 ± 0.00 f	74.36 ± 0.28 bcd	13.13 ± 0.17 gh
S_12_	0.94 ± 0.04 g	0.00 ± 0.00 b	0.00 ± 0.00 d	1.22 ± 0.03 b	7.86 ± 0.11 h	0.00 ± 0.02 g	0.00 ± 0.00 f	74.10 ± 0.24 cd	14.73 ± 0.18 ab
S_13_	2.32 ± 0.22 b	0.26 ± 0.04 a	0.16 ± 0.02 c	0.77 ± 0.04 gh	9.88 ± 0.16 b	0.26 ± 0.04 e	0.26 ± 0.03 d	72.70 ± 0.32 e	12.94 ± 0.13 h
S_14_	1.41 ± 0.06 d	0.00 ± 0.00 b	0.66 ± 0.02 a	0.91 ± 0.03 ef	9.90 ± 0.12 b	1.10 ± 0.05 b	0.33 ± 0.01 b	71.90 ± 0.28 f	12.60 ± 0.16 i
S_15_	1.66 ± 0.06 c	0.00 ± 0.00 b	0.37 ± 0.02 b	0.96 ± 0.01 e	9.90 ± 0.06 b	1.17 ± 0.03 a	0.35 ± 0.01 a	70.80 ± 0.40 g	14.00 ± 0.32 e
IJEC Standard 1998–AOCS [28]	≤3.0	≤1.0	-	-	5.0–15.0	-	-	65.0–80.0	10.0–20.0

The data are presented as the mean ± standard deviation (SD) of three replicates. a–i means with the same letter within the same column are not significantly different according to Duncan’s multiple range test (*p* = 0.05).

**Table 4:** Regions and geographical coordinates of the Egyptian elite jojoba lines.

Lines	Regions	Geographical Coordinates
S_1_	Cairo-Alexandria Desert road	30°06′52.5″ N 30°51′09.9″ E
S_2_	Cairo-Alexandria Desert road	30°06′52.5″ N 30°51′09.9″ E
S_3_	Cairo-Alexandria Desert road	30°06′52.5″ N 30°51′09.9″ E
S_4_	Cairo-Alexandria Desert road	30°06′52.5″ N 30°51′09.9″ E
S_5_	Cairo-Alexandria Desert road	30°06′52.5″ N 30°51′09.9″ E
S_6_	Cairo-Alexandria Desert road	30°06′52.5″ N 30°51′09.9″ E
S_7_	Cairo-Alexandria Desert road	30°06′52.5″ N 30°51′09.9″ E
S_8_	Cairo-Alexandria Desert road	30°06′52.5″ N 30°51′09.9″ E
S_9_	Cairo-Alexandria Desert road	30°06′52.5″ N 30°51′09.9″ E
S_10_	Cairo-Alexandria Desert road	30°06′52.5″ N 30°51′09.9″ E
S_11_	Cairo-Alexandria Desert road	30°06′52.5″ N 30°51′09.9″ E
S_12_	Cairo-Alexandria Desert road	30°06′52.5″ N 30°51′09.9″ E
S_13_	Cairo-Alexandria Desert road	30°06′52.5″ N 30°51′09.9″ E
S_14_	El-Kasasin	30°34′00″ N 31°56′00″ E
S_15_	El-Kasasin	30°34′00″ N 31°56′00″ E

## Data Availability

The data presented in this study are available on request from the corresponding author.

## Sample Availability

Samples of the seeds and jojoba oil are available from the authors.
